# Comparison of Simultaneous Quantitative Analysis of Methylmercury and Inorganic Mercury in Cord Blood Using LC-ICP-MS and LC-CVAFS: The Pilot Study of the Japan Environment and Children’s Study

**DOI:** 10.3390/toxics9040082

**Published:** 2021-04-09

**Authors:** Miyuki Iwai-Shimada, Yayoi Kobayashi, Tomohiko Isobe, Shoji F. Nakayama, Makiko Sekiyama, Yu Taniguchi, Shin Yamazaki, Takehiro Michikawa, Masako Oda, Hiroshi Mitsubuchi, Masafumi Sanefuji, Shouichi Ohga, Nathan Mise, Akihiko Ikegami, Reiko Suga, Masayuki Shimono

**Affiliations:** 1 Japan Environment and Children’s Study Programme Office, National Institute for Environmental Studies, Tsukuba 305-8506, Japan; iwai.miyuki@nies.go.jp (M.I.-S.); kobayashi.yayoi@nies.go.jp (Y.K.); isobe.tomohiko@nies.go.jp (T.I.); sekiyama.makiko@nies.go.jp (M.S.); taniguchi.yu@nies.go.jp (Y.T.); yamazaki.shin@nies.go.jp (S.Y.); 2 Department of Environmental and Occupational Health, School of Medicine, Toho University, Tokyo 143-8540, Japan; takehiro.michikawa@med.toho-u.ac.jp; 3 The Southern Kyusyu and Okinawa Regional Centre, Faculty of Life Sciences, Kumamoto University, Kumamoto 860-8556, Japan; m-oda@kumamoto-u.ac.jp; 4 Department of Neonatology, Kumamoto University Hospital, Kumamoto 860-8556, Japan; mitsubuchi@kuh.kumamoto-u.ac.jp; 5 Research Center for Environment and Developmental Medical Sciences, Kyushu University, Fukuoka 812-8582, Japan; sane26@pediatr.med.kyushu-u.ac.jp (M.S.); ohgas@pediatr.med.kyushu-u.ac.jp (S.O.); 6 Department of Pediatrics, Graduate School of Medical Sciences, Kyushu University, Fukuoka 812-8582, Japan; 7 Department of Environmental and Preventive Medicine, Jichi Medical University, Tochigi 329-0498, Japan; nmise@jichi.ac.jp (N.M.); axi21@jichi.ac.jp (A.I.); 8 Regional Center for Japan Environment and Children’s Study, University of Occupational and Environmental Health, Kitakyushu 807-8555, Japan; rei-suga@med.uoeh-u.ac.jp (R.S.); shimono@med.uoeh-u.ac.jp (M.S.)

**Keywords:** mercury, methylmercury, inorganic mercury, mercury speciation, cohort study, blood, CVAFS, ICP-MS

## Abstract

Prenatal exposure to methylmercury (MeHg) affects child development after birth. However, many epidemiological studies have evaluated total mercury levels without analyzing speciation. Biomonitoring of MeHg and inorganic mercury (IHg) is essential to reveal each exposure level. In this study, we compared a high-throughput analysis for mercury speciation in blood using liquid chromatography-inductively coupled plasma-mass spectrometry (LC-ICP-MS) and liquid chromatography-cold vapor atomic fluorescence spectrometry (LC-CVAFS). The validated LC-ICP-MS method was applied to 101 maternal blood and 366 cord blood samples in the pilot study of the Japan Environment and Children’s Study (JECS). The accuracy of the LC-CVAFS method ranged 90–115% determined by reference material analysis. To evaluate the reliability of 366 cord blood samples, fifty cord blood samples were randomly selected and analyzed using LC-CVAFS. The median (5th–95th percentile) concentrations of MeHg and IHg were 5.4 (1.9–15) and 0.33 (0.12–0.86) ng/mL, respectively, in maternal blood, and 6.3 (2.5–15) and 0.21 (0.08–0.49) ng/mL, respectively, in cord blood. Inter-laboratory comparison showed a relatively good agreement between LC-ICP-MS and LC-CVAFS. The median cord blood:maternal blood ratios of MeHg and IHg were 1.3 and 0.5, respectively. By analyzing speciation, we could focus on the health effects of each chemical form.

## 1. Introduction

Mercury has different toxicokinetics and toxicities depending on its chemical form, which is classified into three types, namely, metallic mercury (or its vapor), organic mercury (methylmercury: MeHg, etc.), and inorganic mercury (IHg). MeHg is a neurotoxic substance and fetal exposure affects child development [1,2,3]. In particular, the concentration of MeHg in cord blood is important for assessing the effect of prenatal exposure on children’s neurodevelopment [1,2,3]. Moreover, microscopic organisms in water and soil can convert IHg into MeHg, leading to accumulation of MeHg in the food chain, particularly in predatory fish [4]. An epidemiological study assessed MeHg exposure by measuring total mercury (THg) in hair and/or blood [2]. Some of the reasons for this are that most people in Japan are exposed to MeHg via fish intake [5,6], most of the mercury in hair and blood is in the form of MeHg, and THg measurements are rapid and inexpensive. However, mercury exposure differs depending on the region and individual. Routes of exposure include gold mining, fish intake, amalgam dental fillings, and injection of vaccines containing thimerosal. Each chemical form of mercury in blood should be measured and its effects should be clarified in humans.

There are two official MeHg analysis methods used in the Ministry of the Environment and the Ministry of Health and Labor and Welfare in Japan. MeHg analysis in the Ministry of the Environment is based on dithizone extraction and gas chromatography coupled with an electron capture detector (GC-ECD), known as the Akagi method [7,8]. This method is exceptionally accurate, and is an analytical method applied in areas where facilities are inadequate, such as in developing countries. However, it is necessary to measure the concentration of each form of mercury, as well as the THg concentration to evaluate the IHg concentration and percentage of MeHg among THg. Due to the development of analytical equipment and technologies, simultaneous quantitative analysis of MeHg and IHg was recently reported using liquid chromatography (LC, (or GC)) coupled with inductively coupled plasma mass spectrometry (ICP-MS) or using LC (or GC) coupled with cold vapor atomic fluorescence spectrometry (CVAFS) [9,10,11,12,13].

We are conducting a large birth cohort study called the Japan Environment and Children’s Study (JECS) [14]. When analyzing 100,000 pairs of samples from mothers and children such as in JECS, it is necessary to use a method that only requires a small amount of a sample, has high sensitivity, and involves simple pretreatment. Therefore, this study aimed to develop a high-throughput method for simultaneous quantitative analysis of MeHg and IHg in human blood. The validated method was then applied in the pilot study of JECS to analyze MeHg and IHg in cord blood and maternal blood. To evaluate the method’s reliability using LC-ICP-MS, we performed an inter-laboratory comparison of MeHg and IHg measurements in blood using LC-ICP-MS and LC-CVAFS. Furthermore, we investigated the relationship between mercury in maternal blood and cord blood.

## 2. Materials and Methods

### 2.1. Study Participants and Blood Collection

**Figure 1:**
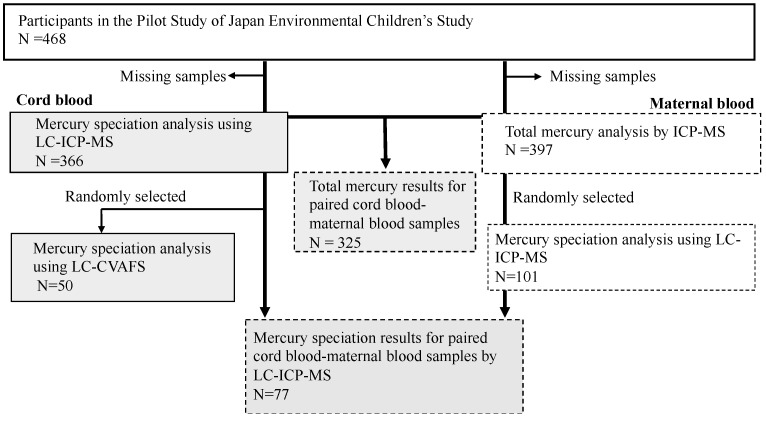
Analytical samples. Boxes in gray, with dotted lines, and in gray with dotted lines indicate cord blood, maternal blood, and paired maternal blood-cord blood samples, respectively. LC: liquid chromatography, ICP-MS: inductively coupled plasma mass spectrometry, CVAFS: cold vapor atomic fluorescence spectrometry.

Participants of the JECS pilot study were registered from February 2009 to March 2010 in four regional centers, namely, Jichi Medical University, University of Occupational and Environmental Health, Kyushu University, and Kumamoto University [15]. For the 453 participants who provided consent, maternal whole blood and cord blood samples were collected in the second to third trimester and at birth, respectively. Mean (standard deviation, SD) of maternal age at birth, gestational weeks, birth weight, and birth height were 31.9 (4.9) years, 38.4 (2.7) weeks, 2988 (445) g, and 49.1 (3.3) cm, respectively. Samples were cooled with ice immediately after collection, frozen at −20 °C, transported to the National Institute for Environmental Studies (NIES), and stored at −80 °C until analysis. Among the collected samples, 366 cord blood and 101 maternal blood samples were available for analysis due to volume limitation (Figure 1). The study was approved by the Ethical Committee in Institutional Review Boards of the National Institute for Environmental Studies (NIES) and four universities (IRB number: 2019-008) on 24 October 2019. Written informed consent was obtained from all individual participants included in the study.

### 2.2. Mercury Speciation Analysis

#### 2.2.1. LC-ICP-MS Analysis

Total 467 (101 maternal and 366 cord blood) samples and reference materials were analyzed for mercury speciation by LC-ICP-MS at a contract laboratory (Osaka, Japan). One-hundred and one maternal blood samples were randomly selected from 366 participants of measured cord blood Hg using statistical software. Samples were packed in dry ice and transported within 24 h. After inspection, the samples were stored at −80 °C until analysis. The analytical method was published elsewhere [16] and is briefly described below.

A standard solution of methylmercury chloride (1000 mg/L) was obtained from Alfa Aesar (Ward Hill, MA, USA). Standard solutions of mercury (II) chloride (1000 mg/L) were obtained from Kanto Chemical Co., Inc. (Tokyo, Japan). Inorganic ^196^Hg was obtained from Cambridge Isotope Laboratories, Inc. (Andover, MA, USA). Standard calibration curves ranged from 0.08 to 39.95 ng/mL for MeHg and from 0.05 to 2.5 ng/mL for IHg (as mercury). Blood samples (0.2 mL) were placed in 1.5 mL polypropylene tubes and mixed with 0.02 mL of a solution containing 1 ng/mL 196 Hg as an internal standard (IS) for IHg. This mixture was incubated at 37 °C with gentle shaking for 5 min. Thereafter, 0.25 mL of 7% (*v*/*v*) hydrochloric acid solution containing 1.5% (*w*/*v*) L-cysteine and 50 ng/mL thallium (Tl) as an IS for MeHg was added. The solution was mixed briefly using a vortex mixer (Figure S1).

Identification and quantification were performed using LC (Agilent 1260 Infinity II Bioinert LC system; Agilent Technologies, Tokyo, Japan) coupled with ICP-MS (Agilent 7900, Agilent Technologies). Chromatographic separation was achieved with a ZORBAX SB-C18 reversed-phase C18 column (Agilent Technologies) at a flow rate of 1.0 mL/min. The instrument settings for LC-ICP-MS are shown in Table 1.

#### 2.2.2. LC-CVAFS Analysis

Fifty cord blood samples were randomly selected from 366 cord blood samples using statistical software. Mercury speciation was performed using LC-CVAFS in our laboratory at the National Institute for Environmental Studies (Tsukuba, Japan).

A standard solution of methylmercury chloride (1000 mg/L) was obtained from Alfa Aesar. Standard solutions of mercury (II) chloride (1000 mg/L) were obtained from Kanto Chemical Co., Inc. The same standard solutions were employed. The standard solution was generated by dilution with 7% (*v*/*v*) hydrochloric acid solution containing 1.5% (*w*/*v*) L-cysteine. Concentrations used to generate the calibration curve ranged from 0.397 to 39.7 ng/mL for MeHg and from 0.50 to 20 ng/mL for IHg. The curves were drawn using nominal concentrations. The pretreatment solution was 7% (*v*/*v*) hydrochloric acid solution containing 1.5% (*w*/*v*) L-cysteine. Blood samples (0.25 mL) were placed and weighted in 1.5 mL polypropylene tubes, and then 0.25 mL of the pretreatment solution was added. After vortexing, the tube was sonicated at room temperature for 30 min (30 cycles, with each cycle comprising sonication for 30 s and a pause for 30 s) using Bioruptor^®^300 (Diagenode Inc., Denville, NJ, USA). After sonication, the tube was centrifuged at 20,400 *g* for 15 min at 4 °C, and ~400 µL of the supernatant was transferred to a new 1.5 mL polypropylene tube. Thereafter, 10% trichloroacetic acid was added and mixed using a vortex mixer. The tube was centrifuged at 20,400× *g* for 5 min, and ~400 µL of the supernatant was transferred to a new 2.0 mL polypropylene tube with a 0.2 µm filter. After centrifugation at 20,400× *g* for 5 min at 4 °C, ~300 µL of the supernatant was transferred to a 0.5 mL glass insert of a 1.5 mL vial. One hundred microliters of the final solution was injected into the LC-CVAFS system (Figures S1 and S2).

Identification and quantification were performed using LC (Prominence LC-20A; Shimadzu, Kyoto, Japan) coupled with CVAFS (PSA 10.025 Millennium merlin 10.025; PSA, Kent, UK). Chromatographic separation was achieved with a Luna 5U C18(2) 100A 50 × 30 mm column (Phenomenex Inc., Torrance, CA, USA) at a flow rate of 0.5 mL/min. The instrument settings for LC-CVAFS are shown in Table 1.

### 2.3. Other Mercury Analysis

THg was measured in maternal blood using ICP-MS, based on the method of Nakayama et al. [17]. To ensure reliability, pooled blood and blood reference materials were also analyzed by both cold vapor atomic absorption spectrometry (CVAAS) for THg and GC-ECD for MeHg. These measurements were performed based on the mercury analysis manual of the Ministry of the Environment, Japan [8].

### 2.4. Quality Control

A linear regression was applied to the calibration curve, and the fitting was evaluated by the coefficient of determination or *r*^2^ for every batch of samples (*r*^2^ > 0.99, Table S1). The batch included solvent blanks, method blanks, 17 standard solutions, reference materials, and blood samples. The method detection limit (MDL) was calculated using data from seven replicated analysis of blood samples that were assumed to have near the minimum concentration in the calibration curve, according to Currie et al. [18] (Table 2). The following formulae was used to calculate the MDL:

MDL = 2 × *s* × *t*_(n−1, 0.05)_ (1)

where *t*_(n−1, 0.05)_ represents Student’s *t* value at an α level of 0.05 with n − 1 degrees of freedom, and s represents the standard deviation (SD).

Analytes were identified with the retention time of each peak. Intra-day and inter-day variations were examined by five replicated analyses of the standard middle concentration (Table 3); relative standard deviation (RSD) was less than 10% in inter-day and intra-day. The trueness of the analytical method was assessed by analyzing reference materials for human blood, which were Seronorm whole blood (level 2) purchased from the Sero AS (Billingstad, Norway) and Quebec blood (PC-B-M 1201, 1203 and 1601) of the Institute National de Santé Publique du Québec (Quebec, QC, Canada). Human red blood cells and plasma were donated by the Japanese Red Cross Society (Tokyo, Japan). Pooled blood was mixed, homogenized, and dispensed into ~2000 tubes in our laboratory. Sample homogeneity was confirmed by analyzing some elements (Table S4). The pooled blood sample and four reference materials were analyzed using different instruments in three laboratories to ensure robustness and reliability. The accuracy of the LC-ICP-MS and LC-CVAFS analyses were within 102–111% and 90–115%, respectively (Tables S2 and S3).

### 2.5. Data Analysis

For right-skewed distributions of mercury concentration data, the median and percentiles are shown in Table 4. The correlations for each mercury concentration were assessed using Spearman’s correlation coefficients (*rho*). Data below the MDL were excluded from analyses. The mercury concentrations for quality control data among institutions were assessed using the non-parametric Wilcoxon Signed Rank (Table 5). *p* < 0.05 was considered statistically significant. All statistical analyses were conducted using JMP 14.0 (SAS Inc., Cary, NC, USA) and R version 4.0.2 (R Foundation for Statistical Computing).

## 3. Results

The validated method of LC-ICP-MS was applied to 101 maternal blood and 366 cord blood samples in the pilot study of JECS (Figure 1). Table 4 shows the concentrations of MeHg and IHg. MeHg was detected in all samples by LC-ICP-MS. The median (5th–95th percentile) concentrations of MeHg and IHg were 5.4 (1.9–15) and 0.33 (0.12–0.86) ng/mL, respectively, in maternal blood and 6.3 (2.5–15) and 0.21 (0.08–0.49) ng/mL, respectively, in cord blood.

Two further analyses using LC-ICP-MS and LC-CVAFS were compared. Pooled blood and three reference materials were used for method validation and quality control. The mean concentrations of MeHg, IHg, and THg were within the reference values provided by Sero AS and the Institute National de Santé Publique du Québec (Table 5). Wilcoxon Signed Rank tests revealed no statistically significant differences between the laboratories for all forms of mercury tested.

**Figure 2:**
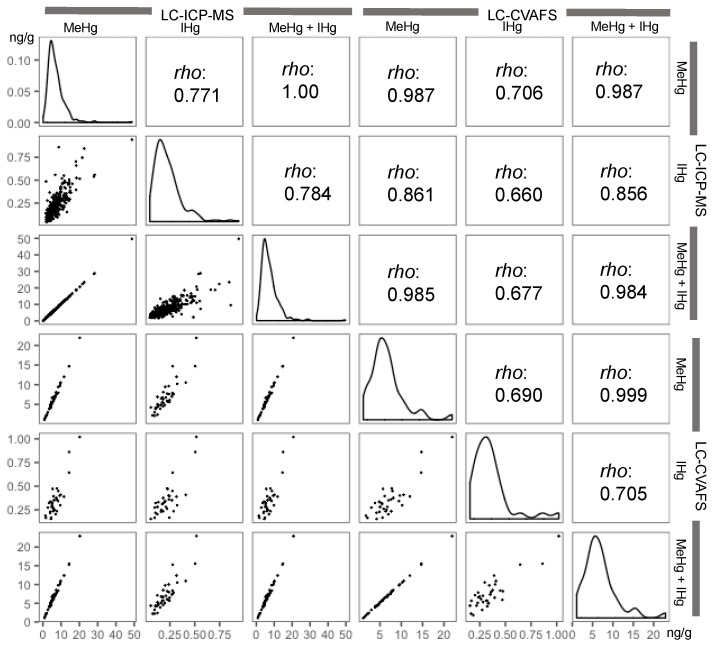
Correlation matrix plots between mercury concentrations (ng/g) in cord blood determined by LC-ICP-MS and LC-CVAFS. Correlation matrix plots show the distribution, ‘*rho*’ of Spearman’s correlation coefficients, and scatter plots. LC-ICP-MS: liquid chromatography-inductively coupled plasma mass spectrometry, LC-CVAFS: liquid chromatography-cold vapor atomic fluorescence spectrometry. The plots of more than method detection limit are shown.

Figure 2 shows the distributions of MeHg and IHg concentrations in cord blood and their sum based on LC-ICP-MS and LC-CVAFS analyses, all of which were right-skewed. Moderate-to-strong correlations between all mercury forms were observed. MeHg and IHg measurements showed relatively good agreement between LC-ICP-MS and LC-CVAFS.

**Figure 3:**
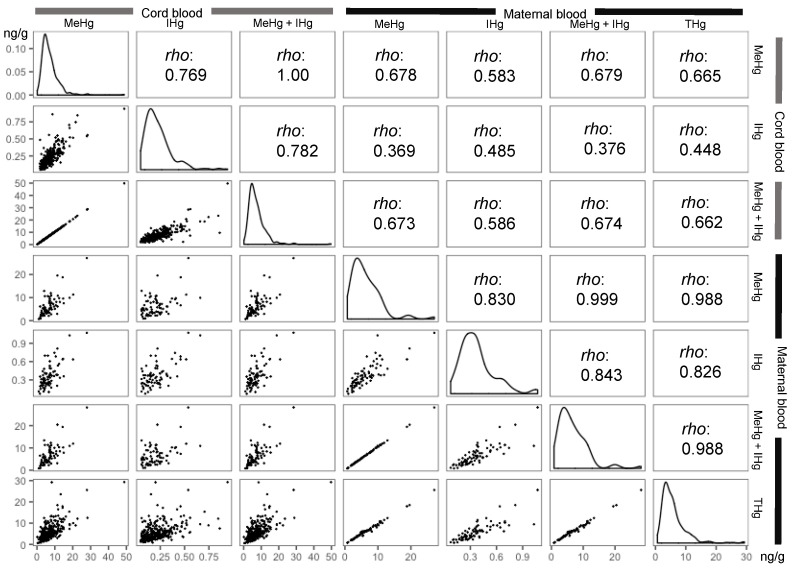
Correlation matrix plots between mercury concentrations (ng/g) in cord blood and maternal blood. Correlation matrix plots show the distribution, ‘*rho*’ of Spearman’s correlation coeffi-cients, and scatter plots. LC-ICP-MS: liquid chromatography-inductively coupled plasma mass spectrometry. Mercury speciation results obtained by LC-ICP-MS are shown as methylmercury (MeHg), inorganic mercury (IHg), and the sum (MeHg + IHg). Total mercury (THg) results ob-tained by ICP-MS are showed as THg. The plots of more than method detection limit are shown.

Figure 3 shows the associations between MeHg concentrations, IHg concentrations, their sums, and THg concentrations in maternal blood and cord blood. There was a strong correlation between MeHg in maternal blood and MeHg in cord blood, but a weak correlation between IHg in cord blood and MeHg in maternal blood.

**Figure 4:**
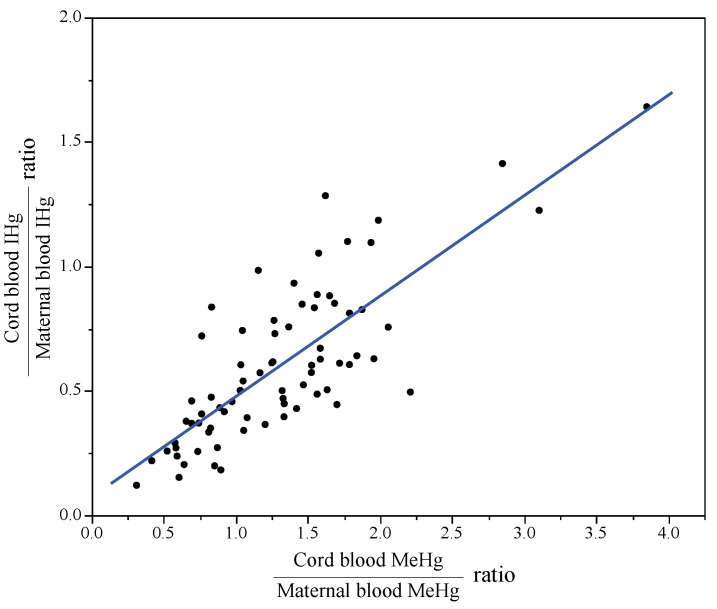
Relationships between cord blood:maternal blood ratios of inorganic mercury (IHg) and methylmercury (MeHg). Y = 0.07 + 0.40 × (*p* < 0.001).

Figure 4 shows the relationships between the cord blood:maternal blood ratios of IHg and MeHg. The median (25th–75th percentile) ratios of MeHg and IHg were 1.3 (0.8–1.6) and 0.5 (0.4–0.8), respectively. The cord blood:maternal blood ratio of IHg was positively associated with that of MeHg.

## 4. Discussions

We developed an alternative method, i.e., LC-CVAFS to the LC-ICPMS for MeHg and IHg in blood and applied it to samples of a birth cohort study. Both LC-ICP-MS and LC-CVAFS involve a rapid pretreatment procedure and can analyze MeHg and IHg simultaneously. LC-ICP-MS can measure low IHg concentrations, while LC-CVAFS can measure higher IHg concentrations. Simultaneous quantitative analysis of MeHg and IHg has been re-ported using LC (or GC) coupled with ICP-MS or using LC (or GC) coupled with CVAFS [9,10,11,12,16,19,20], Table 6). These studies focused on method development and measurements of samples were limited. Wiseman et al. [20] analyzed MeHg and IHg concentrations in blood using isotope dilution (ID)- solid-phase microextraction (SPME)-GC-ICP-MS and applied it to recent immigrant women (N = 76). Our method was applied to samples of a cohort survey, and its analytical validity was verified. By applying this LC-ICP-MS method, 60 samples with calibration standards, blanks, and QC samples can be processed per day, resulting in 1200 samples per month. By LC-CVAFS, 40 samples per day, 800 samples can be analyzed instead. Both had comparable throughput. The LC-CVAFS instrument is five times less expensive than the LC-ICPMS and thus has less hurdle to be used with smaller funding.

The median THg concentration in blood was 0.60 ng/mL in the National Health and Nutrition Survey (NHANES) 2015–2016 (N = 2500, female) of the United States [21] and 3.05 ng/mL in the Korean National Environmental Health Survey (KoNEHS) (N = 6457, [22]). The median THg concentration in maternal blood was 0.64 ng/mL in the MIREC study (N = 1835) of Canada [23] and 3.83 ng/mL in the main study of JECS (N = 17,997, [17]). The median MeHg concentration was 0.40 ng/mL in blood in the NHANES 2013–2014 (N = 2605, female) of the United States [21] and 5.15 ng/g (5.41 ng/mL, adjusted for blood-specific gravity of 1.05) in maternal blood in the Tohoku Study of Child Development (TSCD) of Japan (N = 645, [24]). The median concentration of IHg in maternal blood and cord blood were 0.32 ng/mL (N = 112) and 0.34 ng/mL (N = 98) in Sweden, respectively [25]. The median IHg concentration in blood was less than the limit of detection (0.12 ng/mL) in the NHANES 2013–2014 (N = 2605, female) of the United States [21] and the median IHg concentration in maternal blood was 0.24 ng/g (0.25 ng/mL) in the TSCD of Japan (N = 645, [24]). Our median concentrations of MeHg (5.39 ng/mL) and IHg (0.33 ng/mL) in maternal blood were higher than the median values of female blood in general population of the NHANES. Mercury exposure of the participants was lower in this study than in the Faroese birth cohort study and the Seychelles Child Development Study [2,26,27]. The German Human Biomonitoring Commission (HBM Commission) proclaims health-related guidance values (Human Biomonitoring assessment values, HBM values). The HBM value plays a decisive role in monitoring pollutants and evaluating the exposure of a population, population sub-groups, or individuals. The HBM I value represents no risk of adverse health effects and no need for action. The HBM II value describes above which adverse health effects are possible and an acute need for exposure reduction. The HBM I value and HBM II value of mercury in whole blood for children and adults were 5 μg/L and 15 μg/L, respectively [28]. Half of the participants were between HBM I and HBM II in our study; for its levels, a need for a follow-up study should be performed whether there is continued elevated exposure.

Many studies have reported that MeHg concentrations are higher in cord blood than in maternal blood [24,29,30,31]. We made the same finding. The cord blood:maternal blood ratio of MeHg ranged from 0.8 to 2.8, with a mean value of 1.65 [4]. Ou et al. [29] conducted that Monte Carlo-based meta-analysis to comprehensively estimate the ratios of MeHg and IHg. They reported mean (SD) MeHg and IHg ratios of 1.89 (0.98) and 1.01 (0.55), respectively. These ratios had a log-normal distribution. In this study, the median ratios of MeHg and IHg were 1.3 and 0.5, respectively. The median ratio of MeHg was slightly lower than in previous studies, which might have affected the IHg ratio due to the relationship shown in Figure 4. In general, MeHg and mercury vapor easily cross the placental barrier, whereas IHg is trapped within the placenta [4,32,33]. Inhaled mercury vapor is oxidized to IHg by catalase within the blood [4]. Our ratio results are thought to reflect these findings. We found moderate correlations between MeHg in maternal blood and MeHg in cord blood (Spearman’s *rho* = 0.678), IHg in maternal blood, and IHg in cord blood (*rho* = 0.485). These correlations were mostly consistent with meta-analysis results [29]. Moreover, we consider that the cord blood:maternal blood ratio of IHg was positively associated with that of MeHg. Most of the studies measured MeHg (or IHg) and THg and then subtract MeHg from THg to estimate IHg (or subtract IHg from THg to estimate MeHg) [24,34]. These indirect methods, however, may result in relative uncertainty of the estimate for each Hg form and consequently it would be difficult to make a comparison between studies. The method developed, which can determine MeHg and IHg directly and simultaneously in the single protocol, is suitable for large-scale cohorts and biomonitoring surveys because the uncertainty, sample volume, and cost and time of analysis could be reduced. Although the relationship between the cord blood:maternal blood ratios of MeHg and IHg is a new finding, further investigations are required. Divalent IHg may be bound to metallothionein, which is rich in cysteine, in the placenta [25]. The placenta contains a high level of metallothionein [35]; however, the relationships of metallothionein with mercury have not been well studied in humans.

Most birth cohort studies of mercury analyzed THg and examined its association with effects. Newly developed analytical methods can quantify MeHg and IHg and analyze the exposure level and impact of each chemical form. People who eat fish are mainly exposed to MeHg [2], whereas those who live in gold-mining areas and/or receive amalgam dental fillings have elevated blood IHg levels associated with inhalation exposure to mercury vapor [12]. Some hepatitis B and influenza vaccines contain thimerosal (ethylmercury form). Due to potential exposure to various chemical forms of mercury according to the region and lifestyle of individuals, speciation analysis should be performed as part of large-scale epidemiological studies and human biomonitoring.

There are some limitations of our research. First, the sample size was small; this was a pilot study for JECS, thus the samples were not representative of the Japanese population. However, more detailed findings are expected to be obtained by applying this analytical method to the main study of JECS. Second, there was a time-lag between the collection of maternal blood (second to third trimester) and cord blood (at birth). Lee et al. [36] reported that the intra-class correlation for mercury in whole blood is 0.71. The biological half-lives in blood are about 40–100 days [4,37]. Third, we did not measure ethylmercury in blood. We plan to develop an analytical method for ethylmercury exposure due to vaccination.

## 5. Conclusions

We developed an alternative method, i.e., LC-CVAFS to the LC-ICPMS for MeHg and IHg in blood and applied it to samples of a birth cohort study. LC-ICP-MS and LC-CVAFS require a rapid pretreatment procedure and can analyze MeHg and IHg simultaneously.

## Figures and Tables

**Table 1:** Instrument setting for LC-CVAFS and LC-ICP-MS.

LC-CVAFS	LC	Shimadzu, Prominence LC-20A
	Colum	Luna 5U C18(2) 100A 50 × 30 mm (Phenomenex)
	Mobile phase	Methanol: acetonitrile: ultrapure water (38:30:32, *v*/*v*) + 1.5 mM ammonium pyrrolidinedithiocarbamate
	Flow rate	0.5 mL/min
	Injection volume	100 μL
	Analytical time	10 min
	CVAFS	PSA 10.025 MILLENNIUM MERLIN
	Acid carrier	10% hydrochloric acid + 10% bromine, 2.5 mL/min
	Reductant	2% tin (II) chloride in 10% hydrochloric acid, 4.5 mL/min
	Post collum	UV digestion, 75 °C
LC-ICP-MS	LC	Agilent 1260 Infinity II Bio-inert LC system
	Column	ZORBAX SB-C18, 50 mm × 4.6 mm i.d., 1.8 μm
	Column temperature	15 °C
	Mobile phase	5% (*v*/*v*) methanol, 0.1% (*v*/*v*) 2-mercaptoethanol, and 0.018% (*v*/*v*) hydrochloric acid
	Flow rate	1.0 mL/min
	Injection volume	10 μL
	Cold vapor	0.08% (*w*/*v*) sodium tetrahydroborate in 0.06% (*w*/*v*) sodium hydroxide
	Flow rate	0.2 mL/min
	Valve switching mode	0–3 min: ultrapure water (100%) 3–8.7 min: 0.08% (*w*/*v*) sodium tetrahydroborate (100%) 8.7–10 min: ultrapure water (100%)
	ICP-MS	Agilent 7900
	Spray chamber temperature	2 °C
	Nebulizer gas flow rate	0.66 L/min
	RF power	1600 W
	Plasma gas (Ar) flow rate	15 L/min
	Auxiliary plasma gas (Ar) flow rate	0.90 L/min
	Option gas (20% O_2_ in Ar)	5%
	Isotopes monitored	^202^Hg, ^196^Hg, and ^205^Tl

RF: radio frequency.

**Table 2:** Method detection limit (MDL) calculation.

Low Level Sample	LC-ICP-MS, ng/mL		LC-CVAFS, ng/mL	
	MeHg	IHg	MeHg	IHg
1	0.16	0.12	0.34	0.46
2	0.16	0.11	0.32	0.46
3	0.14	0.10	0.32	0.51
4	0.14	0.10	0.35	0.46
5	0.15	0.11	0.40	0.40
6	0.16	0.11	0.31	0.43
7	0.14	0.11	0.30	0.40
Mean	0.15	0.11	0.33	0.45
SD	0.009	0.006	0.031	0.036
MDL	0.04	0.02	0.12	0.14

MeHg: methylmercury, IHg: inorganic mercury, SD: standard deviation, MDL: method detection limit.

**Table 3:** Reproducibility: repeated measurements of standard middle concentration (ng/mL).

**LC-ICP-MS**	**Sample**	**Run 1**	**Run 2**	**Run 3**	**Run 4**	**Run 5**	**Run 6**	**Run 7**
MeHg	QC-1	3.99	3.93	4.21	4.03	3.58	4.23	3.79
	QC-2	3.79	3.54	3.95	4.19	4.01	4.20	3.93
	QC-3	3.61	3.98	3.92	4.26	4.12	4.23	4.06
	QC-4	3.77	3.85	4.08	3.97	3.95	4.07	4.03
	QC-5	3.92	3.98	4.22	4.28	3.39	3.92	3.91
	RSD (%)	3.8	4.8	3.4	3.4	8.1	3.3	2.7
	RSD Total (%)	5.2						
IHg	QC-1	0.51	0.51	0.48	0.50	0.45	0.54	0.51
	QC-2	0.50	0.48	0.49	0.48	0.43	0.53	0.50
	QC-3	0.50	0.50	0.49	0.52	0.44	0.51	0.50
	QC-4	0.52	0.49	0.50	0.49	0.43	0.51	0.51
	QC-5	0.53	0.49	0.51	0.50	0.45	0.52	0.50
	RSD (%)	2.5	2.3	2.3	3.0	2.3	2.5	1.1
	RSD Total (%)	5.4						
**LC-CVAFS**	**Sample**	**Run 1**	**Run 2**	**Run 3**				
MeHg	QC-1	3.82	3.69	3.84				
	QC-2	3.99	3.71	3.76				
	QC-3	3.87	3.69	3.87				
	QC-4	3.81	3.57	3.89				
	QC-5	3.74	3.74	3.87				
	RSD (%)	2.4	1.8	1.3				
	RSD Total (%)	2.8						
IHg	QC-1	4.90	4.58	5.04				
	QC-2	4.78	4.53	5.06				
	QC-3	4.60	4.63	4.97				
	QC-4	4.79	4.56	4.91				
	QC-5	4.86	5.00	5.03				
	RSD (%)	2.4	4.2	1.2				
	RSD Total (%)	4.0						

MeHg: methylmercury, IHg: inorganic mercury, RSD: relative standard deviation, QC: standard middle concentration.

**Table 4:** Concentrations of methylmercury and inorganic mercury in blood samples from the Japan Environment and Children’s Study (JECS) pilot study.

			N	≥MDL (n)	P5	P25	Median	P75	P95
Cord blood	LC-ICPMS	MeHg (ng/mL)	366	366	2.49	4.40	6.27	9.26	15.1
		(ng/g)	366	366	2.40	4.20	6.04	8.79	14.5
		IHg (ng/mL)	366	355	0.08	0.14	0.21	0.30	0.50
		(ng/g)	366	355	0.08	0.14	0.20	0.28	0.49
	LC-CVAFS	MeHg (ng/mL)	50	50	1.57	4.52	6.26	8.12	14.7
		(ng/g)	50	50	1.56	4.47	6.21	8.15	14.7
		IHg (ng/mL)	50	39	<MDL	0.16	0.29	0.40	0.74
		(ng/g)	50	39	<MDL	0.16	0.29	0.40	0.74
Maternal blood	LC-ICPMS	MeHg (ng/mL)	101	101	1.87	3.25	5.39	8.25	14.7
		(ng/g)	101	101	1.79	3.12	5.18	7.95	14.3
		IHg (ng/mL)	101	101	0.12	0.24	0.33	0.46	0.86
		(ng/g)	101	101	0.11	0.24	0.32	0.44	0.82
	ICPMS	THg (ng/g)	397	397	1.94	3.15	4.66	6.90	13.2

MeHg: methylmercury, IHg: inorganic mercury, THg: total mercury, MDL: method detection limit, P5: 5th percentile. P25: 25th percentile, P75: 75th percentile, P95: 95th percentile.

**Table 5:** Interlaboratory comparison of the concentrations of methylmercury, inorganic mercury, Japan Environment and Children’s Study (JECS and total mercury in blood using reference materials.

		Certified Values (Acceptable Range) (ng/mL)	I: LC-CVAFS (ng/mL)	C: LC-ICP-MS (ng/mL)	A: GC-ECD (ng/g)	CVAAS (ng/g)
			Mean (SD)	Mean (SD)	Mean (SD)	Mean (SD)
Seronorm, Whole blood L2 ^a^	MeHg	1.27 (0.76–1.77)	1.46 (0.14)			
	IHg		14.6 (0.47)			
	THg	17.0 (13.6–20.4)	16.1 (0.60) ^c^			I: 17.0 (0.38) ^e^
Quebec, human blood, PC-B-M1201 ^b^	MeHg		5.11 (0.26)	5.47 (0.07)	5.27(0.05) ^e^	
	IHg		4.62 (0.05)	4.99 (0.09)		
	THg	9.47 (7.04–11.89) ^d^	9.73 (0.23) ^c^	10.5 (0.12) ^c^		A: 8.79 (0.03) ^e^ I: 9.17 (0.09) ^e^
Quebec, human blood, PC-B-M1203 ^b^	MeHg		1.20 (0.17)	1.32 (0.04)	1.20 (0.02) ^e^	
	IHg		0.94 (0.15)	1.14 (0.03)		
	THg	2.37 (1.73–3.01) ^d^	2.14 (0.04) ^c^	2.47 (0.03) ^c^		A: 2.33 (0.05) ^e^ I: 2.44 (0.05) ^e^
Pooled human blood	MeHg		9.46 (0.55)	9.34 (0.31)	8.47 (0.16) ^e^	
	IHg		0.36 (0.12)	0.39 (0.03)		
	THg		9.81 (0.43) ^c^	9.73 (0.30) ^c^		A: 8.97 (0.09) ^e^ I: 9.29 (0.09) ^e^ 9.73 (0.09) ^f^

I: analyzed by Miyuki Iwai-Shimada at the National Institute for Environmental Studies, C: analyzed by a contract company, A: analyzed by the International Mercury Lab. MeHg: methylmercury, IHg: inorganic mercury, THg: total mercury. ^a^ Sero AS (Billingstad, Norway). ^b^ Institute National de Santé Publique du Québec (Quebec, QC, Canada), reference materials with the same levels of MeHg and IHg (50:50) added. ^c^ THg concentrations were calculated as the sums of the MeHg and IHg concentrations. ^d^ The unit of the certified value was converted from nmol/L to ng/mL using the atomic mass of mercury (200.59). ^e^ The results were analyzed based on the mercury analysis manual of the Ministry of Environment of Japan (2004). ^f^ ICP-MS results (see Table S4).

**Table 6:** Summary of speciation mercury analysis of biological samples.

Authors	Year	Samples	Methods	DL/MDL (ng/mL)	Sample Volume (μL)	Pretreatment
Our study	2021	Whole blood	LC-CVAFS	0.12 (MeHg)	250	Acid digestion and sonication
				0.14 (IHg)		
			LC-ICPMS	0.04 (MeHg)	200	
				0.02 (IHg)		
Sogame et al.	2019	Whole blood	LC-ICPMS	0.04 (MeHg) 0.02 (IHg)	200	Acid digestion and sonication
Rodrigues et al.	2010	Whole blood	LC-ICPMS	0.10 (MeHg) 0.15(EtHg) 0.25(IHg)	250	Sonication
de Souza et al.	2013	Plasma	LC-CV-ICPMS	0.004(MeHg) 0.005 (EtHg) 0.012(IHg)	250	Sonication
Baxter et al.	2011	Serum	GC-ID-ICPMS	0.03 (MeHg)	2000	Solvent extraction and derivatization
Brombach et al.	2015	Urine	LC-CVAFS	0.0015 (MeHg)		Microwave
Dórea et al.	2011	Hair	GC-CVAFS	0.5 ng/g (MeHg) 1 ng/g (EtHg)	20 mg	Acid digestion
Wiseman et al.	2019	Whole blood	ID-SPME-GC-ICP-MS	0.16 (MeHg) 0.13 (IHg)	100	TMAH digestion (20 h) and derivatization

MeHg: methylmercury, EtHg: ethylmercury, IHg: inorganic mercury, LC: liquid chromatography, GC: gas chromatography, ICP-MS: inductively coupled plasma mass spectrometry, CVAFS: cold vapor atomic fluorescence spectrometry.

## Data Availability

Data are unsuitable for public deposition due to ethical restrictions and the legal framework in Japan. The Act on the Protection of Personal Information (Act No. 57 passed on 30 May 2003, amended on 9 September 2015) prohibits the public deposition of data containing personal information. Ethical Guidelines for Medical and Health Research Involving Human Subjects enforced by the Japan Ministry of Education, Culture, Sports, Science and Technology and the Ministry of Health, Labour and Welfare also restrict the open sharing of epidemiologic data. All inquiries about access to data should be sent to: jecs-en@nies.go.jp. The person responsible for handling enquiries sent to this e-mail address is Shoji F. Nakayama, JECS Programme Office, National Institute for Environmental Studies.

## Supplementary Materials

The following are available online at https://www.mdpi.com/article/10.3390/toxics9040082/s1, Figure S1: Pretreatment procedures, Figure S2: Chromatographic separation of standard solutions and blood containing methylmercury and inorganic mercury, Table S1: Calibration curve for mercury analysis, Table S2: Repeatability: repeated measurements of concentrations (ng/mL) in reference materials Table S3: Accuracy of measured concentrations (ng/mL) in reference material, Table S4: Homogeneity results of metal concentrations in pooled blood.
