# Supplementary material for: Comparison of Simultaneous Quantitative Analysis of Methylmercury and Inorganic Mercury in Cord Blood Using LC-ICP-MS and LC-CVAFS: The Pilot Study of the Japan Environment and Children’s Study
Source: Toxics. 2021 Apr 9;9(4):82. doi: 10.3390/toxics9040082 (PMC8069649; doi:10.3390/toxics9040082)
Supplement: Supplementary file 1 [file toxics-09-00082-s001.pdf]

# Supplementary Materials: Comparison of Simultaneous Quantitative Analysis of Methylmercury and Inorganic Mercury in Cord Blood Using LC-ICP-MS and LC-CVAFS: The Pilot Study of the Japan Environment and Children's Study

**Miyuki Iwai-Shimada, Yayoi Kobayashi, Tomohiko Isobe, Shoji F. Nakayama, Makiko Sekiyama, Yu Taniguchi, Shin Yamazaki, Takehiro Michikawa, Masako Oda, Hiroshi Mitsubuchi, Masafumi Sanefuji, Shouichi Ohga, Nathan Mise, Akihiko Ikegami, Reiko Suga and Masayuki Shimono**

**This file includes:**

**Figure S1.** Pretreatment procedures

**Figure S2.** Chromatographic separation of standard solutions and blood containing methylmercury and inorganic mercury

**Table S1.** Calibration curve for mercury analysis

**Table S2.** Repeatability: repeated measurements of concentrations (ng/mL) in reference materials

**Table S3**. Accuracy of measured concentrations (ng/mL) in reference materials

**Table S4**. Homogeneity results of metal concentrations in pooled blood

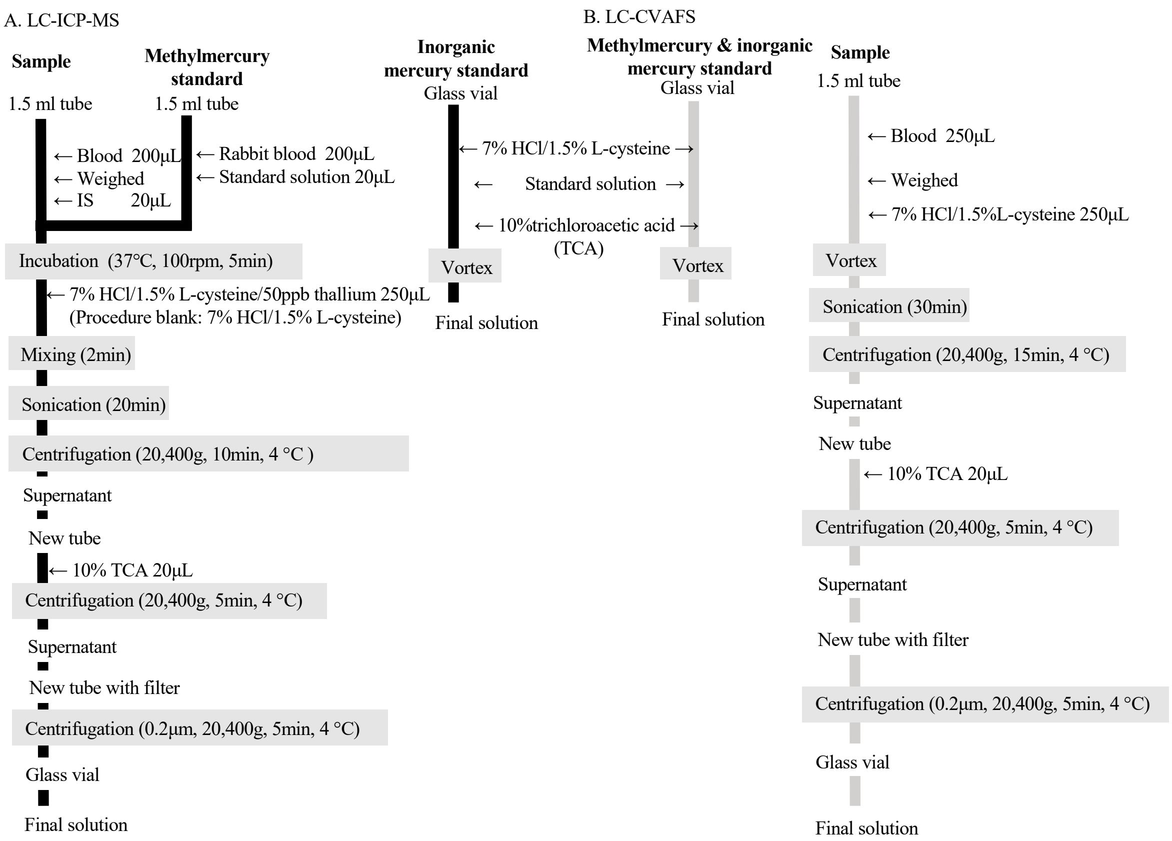


**Figure S1.** Pretreatment procedures. (**A**) and (**B**) showed the pretreatment procedures for LC-ICP-MS and LC-CVAFS, respectively.

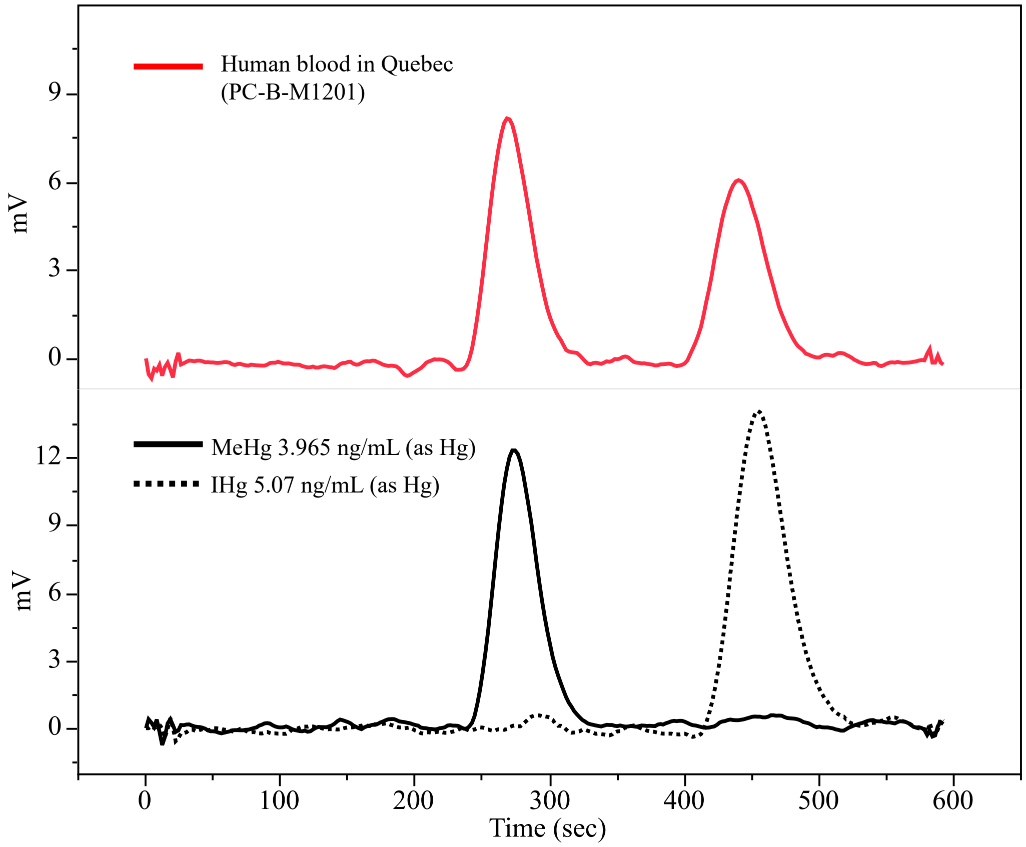


**Figure S2.** Chromatographic separation of standard solutions and blood containing methylmercury and inorganic mercury by LC-CVAFS. Upper, red line: human blood in Quebec (PC-B-M1201). Lower, black line: 3.965 ng/mL methylmercury (MeHg) standard solution (as Hg). Lower, dotted line: 5.07 ng/mL inorganic mercury (IHg) standard solution (as Hg).

**Table S1.** Calibration curve for mercury analysis.

| **Method** | | **Calibration** | |
|---|---|---|---|
| | | **Range (ng/mL)** | ***r*² (min-max)** |
| LC-ICP-MS | Methylmercury | 0.08–39.9 | 0.997–0.999 |
| | Inorganic mercury | 0.05–2.5 | 0.995–0.999 |
| LC-CVAFS | Methylmercury | 0.397–39.7 | 0.999–1.000 |
| | Inorganic mercury | 0.5–20.0 | 0.999–0.999 |

LC-ICP-MS: liquid chromatography-inductively coupled plasma mass spectrometry, LC-CVAFS: liquid chromatography-cold vapor atomic fluorescence spectrometry.

**Table S2.** Repeatability: repeated measurements of concentrations (ng/mL) in reference materials.

| LC-ICP-MS | | Target value (acceptable range) | Run 1 | Run 2 | Run 3 | Run 4 | Run 5 | Run 6 | Run 7 | Run 8 |
|---|---|---|---|---|---|---|---|---|---|---|
| Pooled blood | MeHg | | 8.84 | 9.29 | 9.58 | 9.35 | 9.11 | 9.72 | 9.04 | 9.75 |
| | IHg | | 0.38 | 0.40 | 0.38 | 0.43 | 0.41 | 0.40 | 0.37 | 0.34 |
| | THg | | 9.23* | 9.69* | 9.96* | 9.78* | 9.52* | 10.12* | 9.41* | 10.09* |
| PC-B-M 1601 | MeHg | | 8.61 | 9.47 | 9.21 | 9.92 | 8.74 | 9.85 | 9.44 | 9.80 |
| | IHg | | 1.28 | 1.19 | 1.15 | 1.21 | 1.40 | 1.27 | 1.14 | 1.11 |
| | THg | 10.35 (7.96–12.74) | 9.89* | 10.7* | 10.4* | 11.1* | 10.1* | 11.1* | 10.6* | 10.9* |

| LC-CVAFS | | Target value (acceptable range) | Run 1 | Run 2 | Run 3 |
|---|---|---|---|---|---|
| Pooled blood | MeHg | | 9.96 | 8.87 | 9.54 |
| | IHg | | 0.26 | 0.49 | 0.31 |
| | THg | | 10.22* | 9.36* | 9.85* |
| Seronorm whole blood (Level 2) | MeHg | 1.27 (0.76–1.77) | 1.58 | 1.31 | 1.48 |
| | IHg | | 14.91 | 14.09 | 14.90 |
| | THg | 17.0 (13.6–20.4) | 16.49* | 15.40* | 16.38* |
| PC-B-M1201 | MeHg | | 5.20 | 4.82 | 5.32 |
| | IHg | | 4.65 | 4.64 | 4.56 |
| | THg | 9.47 (7.04–11.9) | 9.85* | 9.46* | 9.88* |
| PC-B-M1203 | MeHg | | 1.35 | 1.02 | 1.23 |
| | IHg | | 0.78 | 1.09 | 0.95 |
| | THg | 2.37 (1.73–3.01) | 2.13* | 2.11* | 2.18* |

*the sum of methylmercury and inorganic mercury. LC-ICP-MS: liquid chromatography-inductively coupled plasma mass spectrometry, LC-CVAFS: liquid chromatography-cold vapor atomic fluorescence spectrometry, RSD: relative standard deviation. The trueness of the analytical method was assessed by analyzing four reference materials of human blood, which were Seronorm whole blood (level 2) and Qubec blood (PC-B-M1601, 1201, and 1203) purchased from Sero AS (Billingstad, Norway) and the Institute National de Santé Publique du Québec (Qubec, Canada). Human red blood cells and plasma for pooled blood were donated by the Japanese Red Cross Society (Tokyo, Japan). *the sum of methylmercury and inorganic mercury. LC-ICP-MS: liquid chromatography-inductively coupled plasma mass spectrometry, LC-CVAFS: liquid chromatography-cold vapor atomic fluorescence spectrometry, RSD: relative standard deviation. The trueness of the analytical method was assessed by analyzing four reference materials of human blood, which were Seronorm whole blood (level 2) and Qubec blood (PC-B-M1601, 1201, and 1203) purchased from Sero AS (Billingstad, Norway) and the Institute National de Santé Publique du Québec (Qubec, Canada). Human red blood cells and plasma for pooled blood were donated by the Japanese Red Cross Society (Tokyo, Japan).

**Table S3.** Accuracy of measured concentrations (ng/mL) in reference materials.

| | | | **Target value (acceptable range)** | **Mean (ng/mL)** | **SD (ng/mL)** | **Accuracy (%)** |
|---|---|---|---|---|---|---|
| LC-ICP-MS | PC-B-M 1601 | MeHg | | 9.38 | 0.50 | |
| | | IHg | | 1.21 | 0.09 | |
| | | THg | 10.35 (7.96–12.74) | 10.6 | 0.45 | 102 |
| | PC-B-M1201 | MeHg | | 5.47 | 0.07 | |
| | | IHg | | 4.99 | 0.09 | |
| | | THg | 9.47 (7.04–11.9) | 10.5 | 0.12 | 111 |
| | PC-B-M1203 | MeHg | | 1.32 | 0.04 | |
| | | IHg | | 1.14 | 0.03 | |
| | | THg | 2.37 (1.73–3.01) | 2.47 | 0.03 | 104 |
| LC-CVAFS | Seronorm whole blood (Level 2) | MeHg | 1.27 (0.76–1.77) | 1.46 | 0.14 | 115 |
| | | IHg | | 14.6 | 0.47 | |
| | | THg | 17.0 (13.6–20.4) | 16.1 | 0.6 | 95 |
| | PC-B-M1201 | MeHg | | 5.11 | 0.26 | |
| | | IHg | | 4.62 | 0.05 | |
| | | THg | 9.47 (7.04–11.9) | 9.73 | 0.23 | 103 |
| | PC-B-M1203 | MeHg | | 1.20 | 0.17 | |
| | | IHg | | 0.94 | 0.15 | |
| | | THg | 2.37 (1.73–3.01) | 2.14 | 0.04 | 90 |

**Table S4.** Homogeneity results of metal concentrations in pooled blood.

| Bottle no. - tube no. | Mn (ng/g) | Se (ng/g) | Cd (ng/g) | Hg (ng/g) | Pb (ng/g) |
|---|---|---|---|---|---|
| 1-1 | 19.1 | 168 | 0.617 | 9.70 | 9.14 |
| 1-2 | 18.7 | 163 | 0.595 | 9.68 | 9.19 |
| 1-3 | 19.0 | 168 | 0.619 | 9.68 | 9.24 |
| 2-1 | 19.0 | 166 | 0.625 | 9.74 | 9.17 |
| 2-2 | 19.0 | 168 | 0.62 | 9.64 | 9.11 |
| 2-3 | 19.0 | 164 | 0.625 | 9.77 | 9.25 |
| 3-1 | 19.2 | 166 | 0.629 | 9.66 | 9.29 |
| 3-2 | 19.2 | 167 | 0.627 | 9.63 | 9.16 |
| 3-3 | 18.8 | 167 | 0.615 | 9.62 | 9.14 |
| 4-1 | 18.7 | 162 | 0.631 | 9.61 | 9.19 |
| 4-2 | 19.2 | 168 | 0.621 | 10.0 | 9.19 |
| 4-3 | 19.0 | 164 | 0.619 | 9.67 | 9.17 |
| 5-1 | 19.0 | 164 | 0.602 | 9.65 | 9.09 |
| 5-2 | 19.0 | 164 | 0.629 | 9.71 | 9.19 |
| 5-3 | 18.8 | 162 | 0.623 | 9.74 | 9.20 |
| 6-1 | 19.0 | 165 | 0.600 | 9.73 | 9.16 |
| 6-2 | 19.0 | 162 | 0.616 | 9.74 | 9.23 |
| 6-3 | 19.0 | 166 | 0.626 | 9.72 | 9.20 |
| 7-1 | 19.1 | 165 | 0.637 | 9.71 | 9.20 |
| 7-2 | 19.3 | 169 | 0.608 | 9.80 | 9.24 |
| 7-3 | 18.8 | 166 | 0.628 | 9.62 | 9.20 |
| 8-1 | 18.9 | 165 | 0.613 | 9.65 | 9.19 |
| 8-2 | 19.1 | 167 | 0.634 | 10.0 | 9.17 |
| 8-3 | 19.3 | 166 | 0.625 | 9.78 | 9.15 |
| 9-1 | 19.5 | 167 | 0.627 | 9.71 | 9.16 |
| 9-2 | 19.0 | 165 | 0.616 | 9.72 | 9.21 |
| 9-3 | 19.2 | 167 | 0.610 | 9.75 | 9.32 |
| 10-1 | 19.2 | 169 | 0.635 | 9.74 | 9.21 |
| 10-2 | 19.0 | 166 | 0.627 | 9.70 | 9.19 |
| 10-3 | 19.1 | 168 | 0.630 | 9.86 | 9.28 |
| 11-1 | 19.0 | 165 | 0.627 | 9.77 | 9.20 |
| 11-2 | 19.2 | 166 | 0.622 | 9.68 | 9.23 |
| 11-3 | 19.0 | 165 | 0.620 | 9.74 | 9.24 |
| 12-1 | 19.0 | 166 | 0.621 | 9.75 | 9.14 |
| 12-2 | 19.2 | 167 | 0.627 | 9.66 | 9.28 |
| 12-3 | 19.2 | 167 | 0.609 | 9.79 | 9.15 |
| Mean | 19.1 | 166 | 0.621 | 9.73 | 9.20 |
| RSD (%) within bottles | 0.80 | 0.94 | 1.46 | 0.73 | 0.53 |
| RSD (%) between bottles | 0.46 | 0.73 | 0.90 | 0.44 | 0.25 |
| F value | 0.743 | 1.41 | 0.957 | 0.640 | 0.527 |
| *P* value | 0.689 | 0.230 | 0.507 | 0.777 | 0.866 |

Mn: manganese, Se: selenium, Cd: cadmium, Hg: mercury, Pb: lead, RSD: relative standard deviation. Human red blood cells and plasma were donated by the Japanese Red Cross Society (Tokyo, Japan). Pooled blood samples were mixed, homogenized, and dispensed into ~2000 tubes in our laboratory. Homogeneity of the pooled blood was confirmed by analyzing five metals. We randomly selected 36 tubes. Metals were measured by ICP-MS, as described elsewhere (Nakayama et al. 2019). A one-way ANOVA revealed no significant differences between bottles.
